# Assessment of Cognitive Impairment in Early Intervention Settings

**DOI:** 10.1192/j.eurpsy.2022.163

**Published:** 2022-09-01

**Authors:** M. Nordentoft, L. Glenthøj, A. Medalia, D. Roberts, C. Hjorthoj

**Affiliations:** 1 CORE-Copenhagen Research Center for Mental Health, Mental Health Center Copenhagen, Hellerup, Denmark; 2 Columbia University Vagelos College of Physicians and Surgeons, New York-Presbyterian, Department Of Psychiatry, New York, United States of America; 3 Division of Community Recovery, Research and Training, University of Texas Health Science Center, Department Of Psychiatry, San Antonio, United States of America

**Keywords:** cognition, RCT, Psychosis, Assessment

## Abstract

Background. Cognitive impairment in schizophrneia is highly prevalent, the level of impairment range from moderate to severe. It has previously beed stated that cognitive impairment was stable through the course of illness, but newer finding from long-term studies indicate that some patient have improved cognitive function. Cognitive function is marginally reactive to antipsychotic medication, and it is highly predictive of poor social and vocational outcome. Also, it constitute a ‘glass ceiling’ for psychosocial and vocational rehabilitation. Several large batteries have been developed, and internationally, there is an attempt to agree on common measurements of core areas. There is a strong rationale for cognitive remediation, namly that it might improve the ability of patients to function in everyday life and that it has no side effects. Individuals at ultra-high risk (UHR) for psychosis have significant cognitive deficits that can impede functional recovery. Methods. In this randomised, clinical trial 146 individuals at UHR for psychosis were randomly assigned to treatment as usual (TAU) or TAU plus cognitive remediation. The CR targeted neurocognitive and social cognitive remediation. Results. A total of 73 UHR individuals were assigned to TAU and 73 assigned to TAU + cognitive remediation. Cognitive remediation did not result in significant improvement on the primary outcome; the Brief Assessment of Cognition in Schizophrenia composite score at 6-month follow-up (b=-0.125, 95%CI: -0.23 to 0.172, p=0.41). Conclusion. The 20-session treatment protocol was not well received in the UHR group. Possibly situations close to everyday life could be better received and be more motivating

**Disclosure:**

No significant relationships.

